# Contribution of Neurochemical Inputs to the Decrease of Motoneuron Excitability During Non-REM and REM Sleep: A Systematic Review

**DOI:** 10.3389/fneur.2018.00629

**Published:** 2018-07-31

**Authors:** Victor B. Fenik

**Affiliations:** ^1^VA Greater Los Angeles Healthcare System, Los Angeles, CA, United States; ^2^Websciences International, Los Angeles, CA, United States

**Keywords:** spinal motoneurons, trigeminal motoneurons, hypoglossal motoneurons, neurotransmitters, genioglossus

## Abstract

The sleep-related depression of excitability of upper airway motoneurons is a major neurological cause of obstructive sleep apnea whereas a disruption in the inhibition of spinal motoneurons during rapid eye movement (REM) sleep causes the REM sleep behavioral disorder. The large amount of experimental data has been obtained that deal with neurochemical mechanisms that are responsible for sleep-related depression of various motoneuron groups. However, there is a disagreement regarding the outcome of these studies primarily due to the use of different animal models and approaches, as well as due to differences in quantification and interpretation of obtained results. In this study, we sought to apply the same calculation methodology in order to uniformly quantify and compare the relative contribution of excitatory or inhibitory inputs to the decrease of excitability of different motoneuronal pools during REM and/or non-REM sleep. We analyzed only published quantitative data that were obtained by using receptor antagonists or chemogenetic approach to block receptors or silence neuronal populations. The outcomes of this analysis highlight the differences in the neurotransmitter mechanisms of sleep-related motoneuron depression between different motoneuronal pools and demonstrate the consistency of these mechanisms for hypoglossal motoneurons among various animal models.

## Introduction

The decrease of upper airway motoneuron excitability during rapid eye movement (REM) sleep and non-REM (NREM) sleep is a major neurological cause of obstructive sleep apnea (OSA), which is recognized as a severe and growing sleep disorder (1, 2). On the other hand, the insufficient inhibition of spinal motoneurons during REM sleep causes REM sleep behavioral disorder (3–5). The OSA is associated with excessive daytime sleepiness, cognitive impairements and decreased quality of life (6–12). The OSA is also linked to hypertension (12–16) and the increased risk of stroke (17). The neurochemical mechanisms that are responsible for REM sleep-related depression of motoneurons have been studied in many laboratories. However, to date, there is no consensus regarding these mechanisms (18–35). The application of receptor antagonists to block neurotransmitter inputs to motoneurons is the main approach in order to assess the involvement of various receptor types and neurotransmitters in the mechanisms of the decrease of motoneuron excitability during both REM sleep and NREM sleep. However, the quantification and interpretation of antagonist effects differed between studies, which contributed to current disagreement of the mechanisms of sleep-related depression of motoneuron activity between various motoneuronal pools.

Recently, a chemogenetic tool was introduced that allows specifically activating or silencing selected groups of neurons by systemic application of clozapine-*N*-oxide (36–38). The chemogenetic activation of A1C1 catecholaminergic neurons was used to study the involvement of these neurons in sleep-related depression of hypoglossal motoneurons (HM) (39).

In this study, we developed an approach that allows quantifying the contribution of excitatory or inhibitory inputs, which were blocked (or removed) by application of receptor antagonists or chemogenetics, to the decrease of motoneuron excitability during NREM and REM sleep. We applied this approach to uniformly assess the contribution of state-dependent inputs to the three groups of motoneurons—spinal, trigeminal, and hypoglossal—for which published quantitative data are available.

## Methodology

**Figure 1:**
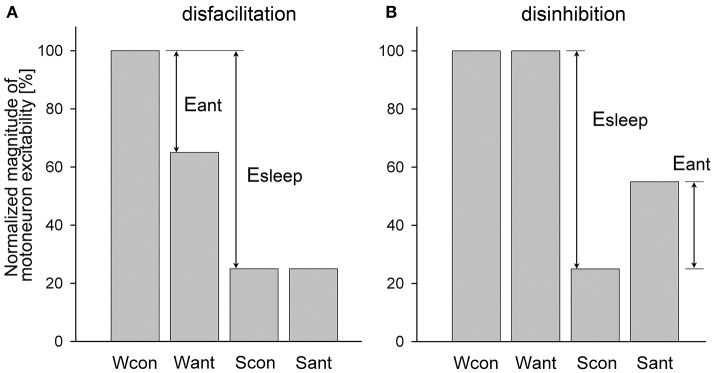
Example of a relatively simple disfacilitatory **(A)** and disinhibitory **(B)** effects of receptor antagonists on motoneuronal activity that blocked only state-dependent input(s) to motoneurons. Wcon and Want, levels of motoneuron activity during wakefulness at the control and after antagonist, respectively; Scon and Sant, levels of motoneuron activity during sleep at control and after antagonist, respectively; Eant and Esleep, effects of antagonist and sleep, respectively.

An example of a relatively simple case when a receptor antagonist application disfacilitated a motoneuronal activity by blocking only excitatory state-dependent input(s) to motoneurons is shown in Figure 1A. During wakefulness, the antagonist application decreased motoneuron activity from the control level (Wcon) to the level after antagonist application (Want). During sleep, the activity reduced to control level (Scon). The level of motoneuron activity after antagonist during sleep (Sant) is equal to Scon because the antagonist already removed the state-dependent excitatory input that would be removed by sleep under control conditions. The relative effect of sleep (Esleep) on the excitability of the motoneurons is calculated as a difference between levels of activity during wakefulness and sleep normalized by Wcon:

(1) $$\begin{array}{r} \text{Esleep}==\left(\text{Wcon}-\text{Scon}\right)/\text{Wco}{\text{n}}^{*}100\% \end{array}$$

The effect of antagonist (Eant) that removes the excitatory input during wakefulness is

(2) $$\begin{array}{r} \text{Eant}==\left(\text{Wcon}-\text{Want}\right)/\text{Wco}{\text{n}}^{*}100\% \end{array}$$

The relative contribution (RC) of the removed excitatory (e) input to the motoneuron excitability compared to the total sleep effect is the following:

$$\begin{array}{l} \text{RCe}==\text{Eant}/\text{Eslee}{\text{p}}^{*}100\%. \end{array}$$

When Equations (1) and (2) are combined, the final equation for the RCe is

(3) $$\begin{array}{r} \text{RCe}==\left(\text{Wcon}-\text{Want}\right)/{\left(\text{Wcon}-\text{Scon}\right)}^{*}100\% \end{array}$$

This example of antagonist action (Figure 1A) is similar to the effect of terazosin, α1-adrenergic receptor antagonist, on the activity of genioglossus muscle during NREM and REM sleep in behaving rats [see Figure 3A in (29)].

An example of the disinhibitory effect of a receptor antagonist is illustrated in Figure 1B. In this example, the antagonist removes only one inhibitory state-dependent input that appears only during sleep. Using the same logic as for the disfacilitation case, the contribution of the removed inhibitory (i) input relative to the total effect of sleep is calculated as following:

(4) $$\begin{array}{r} \text{RCi}==\left(\text{Sant}-\text{Scon}\right)/{\left(\text{Wcon}-\text{Scon}\right)}^{*}100\% \end{array}$$

**Figure 2:**
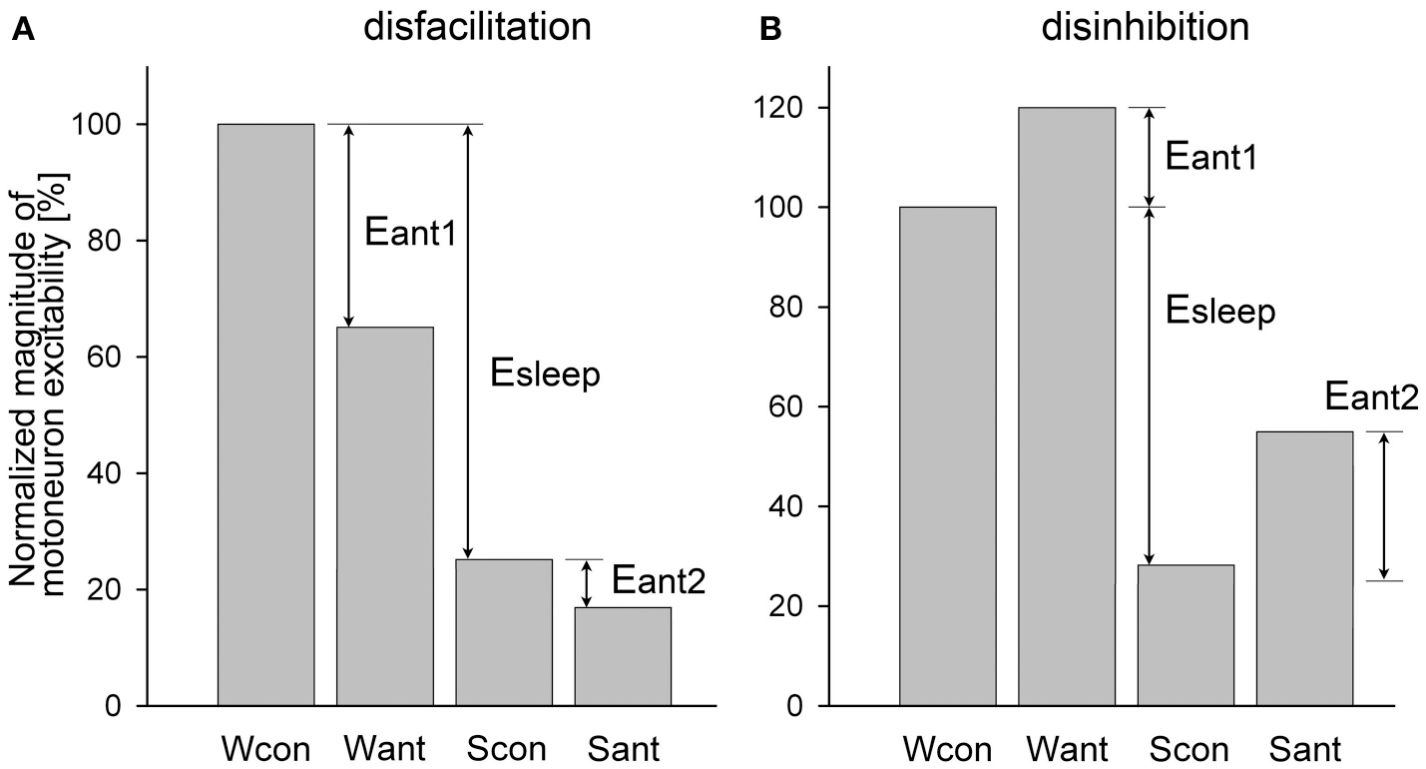
Example of a dual disfacilitatory **(A)** and disinhibitory **(B)** effects of receptor antagonist on motoneuronal activity that blocked both state-dependent and non-state-dependent inputs to motoneurons. Abbreviations are the same as in Figure 1 except for Eant1 and Eant2, which are the effects of antagonists during wakefulness and sleep, respectively.

However, in most experiments, antagonists often block both state-dependent and non-state-dependent inputs to motoneurons, which make it challenging to calculate the magnitude of removed state-dependent input. An example of such dual disfacilitatory effect of an antagonist is shown in Figure 2A. In this example the additional antagonist effect during sleep (Eant2) indicates that the antagonist also removed some non-state-dependent inputs. One approach to qualitatively estimate if antagonist removes any state-dependent input is to compare the relative effects of sleep in control and after antagonist application. For example, if Scon/Wcon < Sant/Want then it would suggest that some state-dependent excitatory input has been removed by antagonist, whereas a condition of Scon/Wcon ≥ Sant/Want would hint that only non-state-dependent input was removed (Figure 2A). Similarly, when the application of an antagonist produces the dual disinhibition, which is apparent by disinhibition of motoneuron activity during both wakefulness and sleep (Figure 2B), the condition of Scon/Wcon ≥ Sant/Want would also indicate that no state-dependent input were removed by antagonist (Figure 2B).

We developed an approach that allows quantifying the relative contribution of a state-dependent input to the total decrease of motoneuron excitability during sleep when receptor antagonist(s) produce the dual effects as discussed above. The approach consists of upgrading the Formulas (3, 4) to include the elimination of the non-state-dependent effects, as following. For the disfacilitatory mixed effect of an antagonist (Figure 2A), i.e., the removal of excitatory state-dependent and excitatory non-state-dependent inputs, the relative contribution of the state-dependent input to depression of motoneuron activity during sleep is

(5) $$\begin{array}{r} \text{RCe}==\left(\text{Wcon}-\text{Wan}{\text{t}}^{*}\text{Scon}/\text{Sant}\right)/{\left(\text{Wcon}-\text{Scon}\right)}^{*}100\% \end{array}$$

The relative contribution of an inhibitory state-dependent input to the sleep-related depression of motoneuron activity for the disinhibitory antagonist mixed effects (Figure 2B), i.e., removal of inhibitory state-dependent and inhibitory non-state-dependent inputs, is calculated as following:

(6) $$\begin{array}{r} \text{RCi}==\left(\text{San}{\text{t}}^{*}\text{Wcon}/\text{Want}-\text{Scon}\right)/{\left(\text{Wcon}-\text{Scon}\right)}^{*}100\% \end{array}$$

Both Formulas (5, 6) work well for either the simple (Figure 1) or the dual (Figure 2) antagonist effects. We applied these formulas to uniformly assess the relative contribution of the removed excitatory or inhibitory state-dependent inputs to the sleep-related decrease of excitability of different motoneuron groups using published data. The contribution to the decrease of motoneuron excitability during NREM sleep was calculated relative to wakefulness, i.e., levels of motoneuron excitability during wakefulness and NREM sleep were used for calculations; whereas the contribution during REM sleep was always assessed relative to NREM sleep, i.e., numbers obtained during NREM sleep and REM sleep were used for the formulas. If the numerical data were not explicitly reported within the text of published manuscripts, we estimated the required numbers from corresponding figures. In cases when Formulas (5, 6) produced numbers that were negative or exceeded 100 (see below), then respectively 0 or 100 were taken as final results.

## Results

### Spinal motoneurons

**Figure 3:**
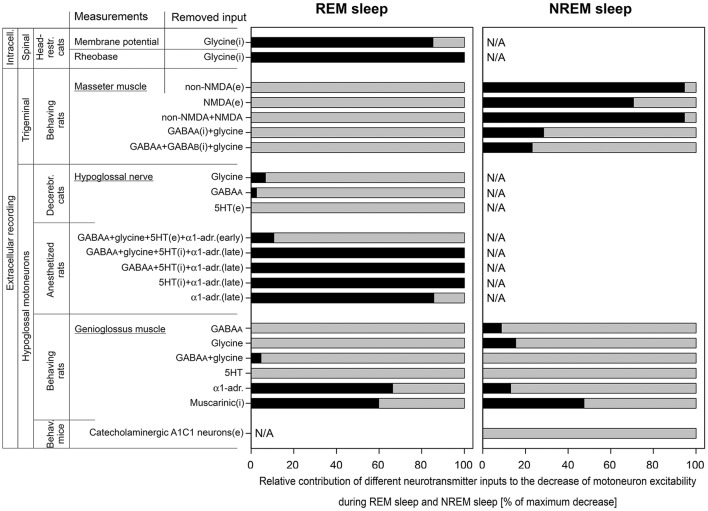
The relative contributions of excitatory (e) and inhibitory (i) inputs to the decrease of motoneuron excitability during REM sleep and NREM sleep that were calculated in this study. Data were used from experiments that were conducted with two approaches: intracellular (intracell.) and extracellular recording; on three motoneuron pools: spinal, trigeminal, and hypoglossal; using different animal models: head-restrained cats, behaving rats, decerebrated cats, anesthetized rats, and behaving mice. The length of black bars shows the relative contribution of tested receptors to the total depression of motoneuron excitability (gray bars) during REM sleep **(left panel)** or NREM sleep **(right panel)**. Note that 5HT behaved as excitatory input in “early” and as inhibitory input in “late” carbachol responses in anesthetized rats.

In the pioneering work conducted by Michael Chase group (40), intracellular recording of lumbar motoneurons were performed during sleep and wakefulness in unanesthetized head-restrained cats. It has been found that the REM sleep-induced decrease of the motoneuron excitability (membrane hyperpolarization and the increase in rheobase) were abolished by strychnine, a glycinergic receptor antagonist, applied iontophoretically to the vicinity of recorded motoneurons. In that study, intracellular parameters of motoneurons were measured during NREM sleep in control (Ncon) and after the antagonist (Nant) and during REM sleep before (Rcon) and after the antagonist (Rant). Corresponding averaged membrane potentials were Ncon = −57.4 mV and Nant = −55.6 mV during NREM sleep and Rcon = −66.5 mV and Rant = −56.9 mV during REM sleep. By the Formula (6), the relative contribution of glycine to the membrane hyperpolarization was RCi = 85.3% (Figure 3). Similarly, for rheobase: Ncon = 9.4 nA; Nant = 9.7 nA; Rcon = 14.9 nA; and Rant = 9.7 nA. The contribution of glycine to the REM sleep-induced increase in rheobase was RCi = 100% (Figure 3).

### Trigeminal motoneurons

The role of glutamatergic, GABAergic, and glycinergic receptors in the decrease of excitability of trigeminal motoneurons during both NREM sleep and REM sleep has been studied in behaving rats (41, 42). Receptor antagonists were applied to the motor trigeminal nucleus using the reverse microdialysis technique and the electromyogram (EMG) of ipsilateral masseter muscle was quantified (41, 42). The glutamatergic antagonists were mostly effective in disfacilitating trigeminal motoneurons during NREM sleep compared to wakefulness but not during REM sleep as compared NREM sleep. For the CNQX, a non-NMDA glutamatergic receptor antagonist, we estimated the following average numbers from Figure 7A in (41), in arbitrary units (AU): Wcon = 47.0; Want = 7.49; Ncon = 5.69; Nant = 5.39; Rcon = 1.35; and Rant = 1.20. Using the Formula (5), we calculated the contribution of non-NMDA receptors RCe = −8.61 = 0% for REM sleep and, for NREM sleep, RCe = 94.6% (Figure 3). Similarly for D-AP5, a NMDA glutamatergic receptor antagonist, we estimated average magnitudes from Figure 7D in (41), in AU: Wcon = 28.3; Want = 13.9; Ncon = 5.93; Nant = 6.60; Rcon = 1.67; and Rant = 1.67. For REM sleep, the calculated contribution was RCe = −15.7 = 0% and, for NREM sleep, RCe = 70.7% (Figure 3). The combined effect of CNQX and D-AP5 was similar to the CNQX effect [Figure 5B in (41)], in AU: Wcon = 37.0; Want = 6.66; Ncon = 5.26; Nant = 5.02; Rcon = 1.33; and Rant = 1.15. For REM sleep, the calculated RCe was −13.9 = 0% and, for NREM sleep, RCe was 94.6% (Figure 3).

The GABAergic and glycinergic antagonists were also more effective in disinhibition of trigeminal motoneurons during NREM sleep than REM sleep. For the effect of combined antagonism of GABA_A_ and glycine receptors on trigeminal motoneurons, we estimated average numbers of masseter EMG from Figure 6B in (42) as following, in AU: Wcon = 2.37; Want = 6.04; Ncon = 0.963; Nant = 3.48; Rcon = 0.664; and Rant = 0.664. Using the Formula (6), for REM sleep, the calculated combined contribution of GABA_A_ and glycinergic receptors RCi = −160 = 0% and, for NREM sleep, RCi was 28.6% (Figure 3). The simultaneous antagonism of GABA_A_, glycine, and GABA_B_ receptors on trigeminal motoneurons produced similar effects on masseter muscle activity. The average numbers of masseter EMG were estimated from Figure 8B in (42), in AU: Wcon = 2.37; Want = 6.96; Ncon = 0.977; Nant = 3.82; Rcon = 0.672; and Rant = 1.00. For REM sleep, the calculated RCi = −136 = 0% and, for NREM sleep, RCi = 23.2% (Figure 3).

### Hypoglossal motoneurons

The decrease of excitability of HM during NREM and REM sleep was investigated in several studies using various animal models. The increased interest to HM was mainly due to their innervation of upper airway muscles including the genioglossus, which play a critical role in maintaining the upper airway patency in OSA patients (43–46).

In early studies, the REM sleep-related decrease of excitability of HM was studied in a quantitative manner using decerebrated cats and anesthetized rats (19, 25). In both animal models, the REM sleep-like state was triggered by microinjections of carbachol, a cholinergic agonist, into dorsolateral pontine tegmentum and receptor antagonists were microinjected into the hypoglossal motor nucleus while spontaneous inspiratory activity of HM was recorded and quantified in ipsilateral hypoglossal nerve. In decerebrated cats, antagonizing GABA_A_ and glycinergic receptors within the hypoglossal nucleus disinhibited HM during the baseline NREM sleep-like condition. The averaged normalized values of hypoglossal nerve activity during antagonism of glycinergic receptors (19) were the following, in %: Ncon = 107; Nant = 132; Rcon = 14; and Rant = 25. By the Formula (6), the contribution of glycine to the decrease of excitability of HM during REM sleep-like state was RCi = 6.74% (Figure 3). Similarly, for the antagonism of GABA_A_ receptors (19), the hypoglossal nerve activity was, in %: Ncon = 69; Nant = 138; Rcon = 10.5; and Rant = 24. The calculated RCi was 2.56% (Figure 3). The contribution of serotonergic (5HT) effects to carbachol-induced depression of HM activity has been studied with methysergide, a broad-spectrum 5HT antagonist, that decreased HM activity (47). For the calculations, we used the following averaged normalized magnitudes of hypoglossal nerve activity, in %: Ncon = 100; Nant = 54; Rcon = 10; and Rant = 5. By the Formula (5), the contribution of 5HT receptors to the reduction of HM excitability during carbachol-induced REM sleep-like state was calculated as RCe = −8.89 = 0% (Figure 3).

In the REM sleep model using anesthetized rats, pontine carbachol could repeatedly elicit REM sleep-like state in the same animals, which helped to study neurochemical mechanisms of the depressant effect of REM sleep on HM excitability. In one study, a mix containing four antagonists—bicuculline, strychnine, methysergide, and prazosin—to antagonize GABA_A_, glycine, 5HT and α1-adrenergic receptors, respectively, was microinjected into hypoglossal nucleus (25). Early after the injections, the spontaneous inspiratory activity in ipsilateral hypoglossal nerve was disinhibited. Averaged normalized amplitudes of the nerve activity were estimated from the Figure 3B in (25) and were the following, in %: Ncon = 100; Nant = 140; Rcon = 18.6; and Rant = 38.2. The calculated RCi for the “early” effect by the Formula (6) was 10.7% (Figure 3). In 30–60 min after the injection of antagonists, hypoglossal nerve activity was disfacilitated and decreased below the control level most likely due to diffusion of the antagonists (26), which led to the abolition of the carbachol-induced depression of the nerve activity. The average nerve activity for these “late” carbachol trials was estimated from the Figure 3B in (25) as following, in %: Ncon = 100; Nant = 40; Rcon = 18.6; and Rant = 46.8. By the Formula (5), the calculated RCe for the “late” effect was 103 = 100% (Figure 3).

The abolition of the carbachol-induced depression of HM that occurred ~30 min after microinjections of the four antagonists into hypoglossal nucleus prompted us for additional studies to determine the role of each antagonist in this effect (26, 27). Microinjections of three antagonists to simultaneously antagonize GABA_A_, 5HT,and α1-adrenergic receptors resulted in the following numbers of the average normalized nerve activity for the “late” antagonist mix effect (26), in %: Ncon = 100; Nant = 32.8; Rcon = 28.6; and Rant = 37.7 (32.8^*^1.15). By the Formula (5), the calculated RCe was 105 = 100% (Figure 3). When only two monoaminergic 5HT and α1-adrenergic receptors were simultaneously antagonized, the depression of HM during REM sleep-like state was also abolished (27), in %: Ncon = 100; Nant = 27.0; Rcon = 24.9; and Rant = 25.2. The calculated RCe for the “late” antagonist mix effect was 100% (Figure 3). Further single injections of antagonists proved that the simultaneous blocking of 5HT and α1-adrenergic receptors were required for the abolition effect, while the separate antagonism of either α1-adrenergic or 5HT receptors was insufficient. The corresponding numbers for the “late” effect of the antagonism of α1-adrenergic receptors were the following (27), in %: Ncon = 100; Nant = 20.8; Rcon = 26.1; and Rant = 14.8. The RCe calculated by the Formula (5) was 85.7% (Figure 3). Antagonizing only 5HT receptors had moderate effect on carbachol-induced depression of HM activity. In addition, the methysergide effect was complicated by changes of the activity of HM that had opposite directions. Early after methysergide injection, the HM activity was disfacilitated for a long time. However, approximately after 30 min, the HM activity was disinhibited on the top of the long-lasting disfacilitation that started earlier [Figure 3C in (27)]. This mixed effect of methysergide was masked by a stronger effect of blocking α1-adrenoceptors by prazosin when it was present in the antagonist mix. Due to the opposite effects of methysergide, either Formula (5, 6) could not be used for the calculation of 5HT contribution. However, the disinhibitory contribution of 5HT receptors during the “late” effect could be estimated as difference between the full REM sleep-like depression of HM activity and the effect of α1-adrenergic receptors (see above) as following: 100 – 85.7 = 14.3%.

Important data has been obtained during natural sleep and wakefulness in behaving rats that has greatly advanced our understanding of the mechanisms of sleep-related depression of HM (28, 29, 33, 48, 35). The reverse microdialysis was used to deliver antagonists into the hypoglossal nucleus and the EMG of the genioglossus (GG) muscle was quantified to assess the antagonist effects during sleep and wakefulness. Bicuculline and strychnine were used to antagonize GABA_A_ and glycine receptors, respectively (48, 35). For the effect of bicuculline, we estimated the average magnitude of respiratory EMG of GG at room air using Figure 4 in (48), in AU: Wcon = 18.4; Want = 54.4; Ncon = 3.61; Nant = 14.5; Rcon = 1.35; and Rant = 2.03. The calculated relative contribution of GABA_A_ receptors the sleep-related depression of GG EMG by the Formula (6) was RCi = −37.4 = 0% for REM sleep and RCi = 8.75% for NREM sleep (Figure 3). For the antagonizing glycinergic receptors by strychnine, the average magnitude of respiratory activity of GG at room air was estimated from Figure 4 in (35), in AU: Wcon = 35.2; Want = 54.2; Ncon = 7.29; Nant = 17.9; Rcon = 1.10; and Rant = 2.34. The calculated relative contribution of glycinergic receptors was RCi = −2.37 = 0% for REM sleep and RCi = 15.5% for NREM sleep (Figure 3). For the combined effect of bicuculline and strychnine, numbers of the average magnitude of respiratory activity of the GG muscle were estimated from Figure 8 in (35), in AU: Wcon = 25.8; Want = 101; Ncon = 6.52; Nant = 17.1; Rcon = 1.12; and Rant = 3.60. The relative contribution of combined GABA_A_ and glycine receptors was RCi = 4.68% for REM sleep and RCi = −11.2 = 0% for NREM sleep (Figure 3).

The effect of 5HT receptor antagonism on the GG muscle EMG have been studied using mianserin, a broad-spectrum 5HT antagonist (28). There was no visible effect of mianserin in the room air trials during NREM sleep or REM sleep and both Formulas (5, 6) produced negative results; therefore, zeros were entered in Figure 3.

Terazosin was used to study the role of α1-adrenoceptors in sleep-related decrease of GG activity in behaving rats (29). The disfacilitatory effect of terazosin was assessed using estimated numbers of the average magnitude of respiratory EMG of the GG muscle from Figure 3A in (29), in AU: Wcon = 44.2; Want = 24.5; Ncon = 29.7; Nant = 17.2; Rcon = 7.74; and Rant = 8.80. By the Formula (5), the relative contribution of α1-adrenergic receptors was RCe = 66.4% for REM sleep and RCe = 13.1% for NREM sleep (Figure 3).

The involvement of muscarinic receptor in the decrease of the GG muscle activity during NREM and REM sleep has been studied with scopolamine, a broad-spectrum muscarinic receptor antagonist (33). The antagonist had a disinhibitory effect on the GG muscle and we estimated numbers for average respiratory GG muscle activity during wakefulness, NREM sleep and REM (–) sleep from Figure 1D in (33), in AU: Wcon = 22.7; Want = 26.6; Ncon = 9.06; Nant = 18.2; Rcon = 0; and Rant = 10.9. By the Formula (6), the relative contribution of muscarinic receptors was RCi = 59.9% during REM (–) sleep and RCi = 47.5% for NREM sleep (Figure 3).

The role of A1C1 catecholaminergic neurons in control of HM has been studied using chemogenetics in behaving transgenic mice. Inhibitory DREADD (designer receptors activated by designer drug) has been used to study the effect of inhibition of A1C1 neurons on EMG of the GG muscle during NREM sleep and wakefulness (39). The silencing of A1C1 catecholaminergic neurons disfacilitated the GG muscle activity with the following numbers for averaged magnitude of GG EMG, in AU: Wcon = 142; Want = 120; Ncon = 37.2; Nant = 28.1. The relative contribution of A1C1 neurons to the GG muscle depression during NREM sleep calculated by Formula (5) was RCe = −16.1 = 0% (Figure 3).

## Discussion

The major advantage of our analysis is that the single approach was used to uniformly quantify the contribution of excitatory and inhibitory inputs to the sleep-induced decrease of motoneuron excitability in different motoneuron pools. The relative contribution of state-dependent excitatory inputs to the total depressing effect of sleep was calculated using the Formula (5) whereas the contribution of state-dependent inhibitory inputs was calculated using Formula (6). These formulas can produce reliable results only when both state-dependent and non-state-dependent inputs that are removed by antagonists are of the same type, either excitatory or inhibitory; otherwise, the formulas produce false results. For example, methysergide had both disfacilitatory and disinhibitory effects on HM activity at the same time [see Figure 7A in (27)] and therefore we could not use either formula. In addition, the variability of data greatly affected the results of both formulas, which often exceeded the expected range of results 0–100%.

The performed analysis of the state-dependent inputs, which contribute to the decrease of excitability of motoneurons during NREM and REM sleep, reveals that the neurochemical mechanisms of sleep-induced motoneuron depression is considerably different between the three motoneuronal pools (Figure 3). The major points of this finding are the following. (1) In agreement with the author conclusion (40), our calculations indicate that the spinal motoneurons are inhibited by ~100% by glycine during REM sleep (depression of the motoneurons was not studied during NREM sleep in this study). (2) Our analysis suggests that the trigeminal motoneurons are not inhibited by either GABA or glycine, nor disfacilitated by glutamatergic mechanisms, during REM sleep. Although, the glutamatergic disfacilitation plays a major role in depression of trigeminal motoneurons during NREM sleep while either GABA_A_ or glycine receptors, or both, have a moderate contribution to NREM sleep-induced depression of trigeminal motoneurons (41, 42). (3) Our analysis also suggests the following: (a) However, both GABA and glycine may play a role during NREM sleep-induced depression of HM (19, 25, 27, 28, 47, 48, 35); (b) Noradrenergic excitatory input plays a major role in HM depression during REM sleep-like state in anesthetized rats (27); (c) However, during natural REM sleep, noradrenergic and muscarinic mechanisms contribute ~50/50% to the depression of HM activity (29, 33). In addition, our analysis revealed some contribution of noradrenergic disfacilitation and approximately 50% of muscarinic inhibition during natural NREM sleep-induced depression of HM (29, 33); (d) Our analysis also confirmed no contribution of A1C1 neurons to HM depression during NREM sleep as was concluded by authors (39).

## Conclusion

Our calculations confirmed that during natural REM sleep the decrease of excitability of spinal motoneurons is mediated only by glycinergic receptors (100%). The depression of trigeminal motoneurons during NREM sleep could be fully explained by excitatory glutamatergic input (~70%) and inhibitory GABAergic and/or glycinergic inputs (30%) whereas the contribution of these inputs during REM sleep was not detected by using either Formulas (5, 6). The depression of HM during natural REM sleep is approximately equally mediated by α1-adrenoceptors and muscarinic receptors (50/50%). In anesthetized animal model of REM sleep, α1-adrenoceptors mainly contributed to depression of HM during the “late” carbachol-induced REM sleep-like episodes (with some 5HT receptor contribution), which together fully (100%) accounted for the depressant effect of REM sleep-like state on HM. During the “early” carbachol-induced REM sleep-like episodes, the combined GABA_A_, glycinergic, 5HT, and α1-adrenergic inputs minimally contributed to the depression of HM. Other inputs must be responsible for HM depression during “early” REM sleep-like episodes, e.g., muscarinic, but this possibility was not tested. The neurotransmitters GABA, glycine and 5HT had minimal or no contribution to REM sleep-induced depression of HM activity regardless of used animal model. The contribution of A1C1 catecholaminergic neurons to NREM sleep-related depression of HM was not detected using the Formula (5).

## Author contributions

VF developed the calculation approach, quantitatively analyzed available published data and wrote the manuscript.

### Conflict of interest statement

The author declares that the research was conducted in the absence of any commercial or financial relationships that could be construed as a potential conflict of interest.
